# Lurbinectedin, a selective inhibitor of oncogenic transcription, in patients with pretreated germline *BRCA1/2* metastatic breast cancer: results from a phase II basket study

**DOI:** 10.1016/j.esmoop.2022.100571

**Published:** 2022-08-28

**Authors:** V. Boni, B. Pistilli, I. Braña, G.I. Shapiro, J. Trigo, V. Moreno, D. Castellano, C. Fernández, C. Kahatt, V. Alfaro, M. Siguero, A. Zeaiter, F. Longo, K. Zaman, A. Antón, A. Paredes, G. Huidobro, V. Subbiah

**Affiliations:** 1 START Madrid-CIOCC, Centro Integral Oncológico Clara Campal, Madrid, Spain; 2 Gustave Roussy, Villejuif, France; 3 Hospital Universitario Vall D'Hebron (VHIO), Barcelona, Spain; 4 Dana-Farber Cancer Institute, Boston, USA; 5 Hospital Universitario Virgen De La Victoria, IBIMA, Málaga, Spain; 6 START Madrid-FJD, Hospital Universitario Fundación Jiménez Díaz, Madrid, Spain; 7 Hospital Universitario 12 de Octubre, Madrid, Spain; 8 PharmaMar, Colmenar Viejo, Madrid, Spain; 9 Hospital Universitario Ramón y Cajal, Madrid, Spain; 10 University Hospital CHUV, Lausanne, Switzerland; 11 Hospital Universitario Miguel Servet, Zaragoza, Spain; 12 Hospital Universitario Donostia, Donostia-San Sebastián, Spain; 13 Hospital Universitario de Vigo Alvaro Cunqueiro, Pontevedra, Spain; 14 The University of Texas MD Anderson Cancer Center, Houston, USA

**Keywords:** lurbinectedin, breast cancer, *BRCA1*, *BRCA2*, response rate, phase II

## Abstract

**Background:**

Lurbinectedin, a selective inhibitor of oncogenic transcription, has shown preclinical antitumor activity against homologous recombination repair-deficient models and preliminary clinical activity in *BRCA*1/2 breast cancer.

**Patients and methods:**

This phase II basket multitumor trial (NCT02454972) evaluated lurbinectedin 3.2 mg/m^2^ 1-h intravenous infusion every 3 weeks in a cohort of 21 patients with pretreated germline *BRCA*1/2 breast cancer. Patients with any hormone receptor and human epidermal growth factor receptor 2 status were enrolled. The primary efficacy endpoint was overall response rate (ORR) according to RECIST v1.1. Secondary endpoints included duration of response (DoR), progression-free survival (PFS), overall survival (OS) and safety.

**Results:**

Confirmed partial response (PR) was observed in six patients [ORR = 28.6%; 95% confidence interval (CI) 11.3% to 52.2%] who had received a median of two prior advanced chemotherapy lines. Lurbinectedin was active in both BRCA mutations: four PRs in 11 patients (36.4%) with *BRCA*2 and two PRs in 10 patients (20.0%) with *BRCA*1. Median DoR was 8.6 months, median PFS was 4.1 months and median OS was 16.1 months. Stable disease (SD) was observed in 10 patients (47.6%), including 3 with unconfirmed response in a subsequent tumor assessment [ORR unconfirmed = 42.9% (95% CI 21.8% to 66.0%)]. Clinical benefit rate (PR + SD ≥ 4 months) was 76.2% (95% CI 52.8% to 91.8%). No objective response was observed among patients who had received prior poly (ADP-ribose) polymerase inhibitors. The most common treatment-related adverse events (AEs) were nausea (61.9%), fatigue (38.1%) and vomiting (23.8%). These AEs were mostly grade 1/2. The most common grade 3/4 toxicity was neutropenia (42.9%: grade 4, 23.8%: with no febrile neutropenia).

**Conclusions:**

This phase II study met its primary endpoint and showed activity of lurbinectedin in germline *BRCA*1/2 breast cancer. Lurbinectedin showed a predictable and manageable safety profile. Considering the exploratory aim of this trial as well as previous results in other phase II studies, further development of lurbinectedin in this indication is warranted.

## Highlights

• Loss of function due to mutations in the breast cancer gene, *BRCA*, leads to worse prognosis in breast cancer patients.
• Lurbinectedin has previously shown antitumor activity in breast cancer patients with mutations in *BRCA2.*
• This phase II basket study included a cohort of 21 patients with *BRCA*-mutated breast cancer treated with lurbinectedin.
• The study showed antitumor activity and supports further investigation in this type of cancer.

## Introduction

Breast cancer with *BRCA1/2* mutations is emerging as a distinctive group of breast cancers that present at an earlier age with hallmarks of genomic instability and accumulation of DNA damage.^1^, ^2^, ^3^ Two poly (ADP-ribose) polymerase inhibitors (PARPi) are available as the therapeutic option (olaparib and talazoparib), but many patients do not derive benefit because of multiple primary and secondary resistance mechanisms and toxicities.^4^ Novel class of agents are needed to be developed beyond the current PARPi or the central protein kinases ataxia telangiectasia mutated (ATM) and ataxia telangiectasia and Rad3-related (ATR) inhibitors.

Lurbinectedin is a selective inhibitor of oncogenic transcription that leads to cell apoptosis^5^ and also inhibits activated transcription in tumor-associated macrophages.^6^ Lurbinectedin has antitumor activity against homologous recombination repair-deficient (HRD) cell lines.^7^^,^^8^ The mechanism involves the irreversible stalling of elongating RNA polymerase II (Pol II) on the DNA template and its specific degradation by the ubiquitin/proteasome machinery. Subsequently, recruitment of DNA repair factors including xeroderma pigmentosum complementation group F nuclease induces the accumulation of double-strand breaks and apoptosis as downstream events.^9^ These effects are increased in HRD cells. In fact, in *BRCA2*-mutated cells, this could be related to the concurrence of deficient DNA repair and formation of R-loops that occurs during the elongation step of transcription by RNA polymerase II.^10^^,^^11^

In a basket, open-label, phase II study (ClinicalTrials.gov identifier: NCT02454972), nine cohorts of patients with different tumor types were treated with lurbinectedin. Based on the results in the small-cell lung cancer (SCLC) cohort,^12^ approval of lurbinectedin was obtained in this indication first in the United States^13^ and later in other countries (Canada, Australia, Singapore and Arab Emirates). This report focuses on the outcomes in the germline *BRCA*1/2 breast cancer cohort. This cohort was evaluated because, in a previous phase II study, lurbinectedin had shown antitumor activity in patients with advanced breast cancer and germline *BRCA*1/2 pathogenic variants: overall response rate (ORR) was 41% and median overall survival (OS) was 20.0 months compared to 9% and 12.5 months, respectively, in patients with *BRCA*1/2 wild-type or unknown status.^14^

## Methods

The study protocol was approved by the independent local ethics committee of each participating center and was conducted in accordance with the Declaration of Helsinki, Good Clinical Practice guidelines and local regulations for clinical trials. Signed informed consent was obtained from all patients before their inclusion in the study.

### Patient selection

Twenty-one patients with germline *BRCA*1/2 breast cancer were treated at 12 investigational sites in France (*n* = 3), Spain (*n* = 15), Switzerland (*n* = 1) and the United States (*n* = 2). Eligibility criteria included patients ≥ 18 years old with pathologically proven diagnosis of germline *BRCA*1/2 metastatic breast carcinoma; pretreated with one to three chemotherapy-containing lines [prior endocrine and/or human epidermal growth factor receptor 2 (HER2)-directed treatment were allowed]; measurable disease as per RECIST v.1.1;^15^ Eastern Cooperative Oncology Group performance status ≤ 2; and adequate major organ function (including neutrophil count ≥ 2.0 × 10^9^/l). Patients were excluded if they had: previously received lurbinectedin or trabectedin; prior or concurrent malignant disease unless in complete remission for >5 years; known central nervous system involvement (active or stable/treated disease); concomitant unstable or serious medical condition; or an impending need for radiotherapy.

### Lurbinectedin treatment

All patients were treated with lurbinectedin 3.2 mg/m^2^ administered as a 1-h intravenous (i.v.) infusion every 3 weeks (q3wk). All patients received antiemetic prophylaxis. Primary granulocyte colony-stimulating factors (G-CSFs) prophylaxis was not allowed.

### Assessments

The primary objective of this study was to assess the antitumor activity of lurbinectedin in terms of ORR, the primary endpoint, as assessed by the investigators. Radiological tumor evaluation was carried out every 6 weeks (two cycles) until cycle 6, and every 9 weeks (three cycles) thereafter. Objective response was to be confirmed at least 4 weeks later. Secondary efficacy endpoints included disease control rate (ORR or stable disease), duration of response (DoR), progression-free survival (PFS) and OS.

Safety was evaluated in all patients who received at least one lurbinectedin infusion, complete or incomplete, by assessment of adverse events (AEs), clinical laboratory test results, physical examinations and vital signs. Laboratory tests were done weekly during cycles 1 and 2, and on day 1 of subsequent cycles. AEs were recorded and coded with the Medical Dictionary for Regulatory Activities (MedDRA) v.21.0. AEs and laboratory values were graded according to the National Cancer Institute-Common Toxicity Criteria for Adverse Events (NCI-CTCAE) v. 4.0. All patients were followed until recovery from any lurbinectedin-related AE.

### Statistical methods

Up to 25 patients were to be recruited to test the null hypothesis that 1% or fewer patients would have a response (*P* ≤ 0.01) versus the alternative hypothesis that 10% or more patients would have a response (*P* ≥ 0.10). The variance of the standardized test was based on the null hypothesis. The type I error (alpha) associated with this one-sided test is 0.025 and the type II error (beta) is 0.2; hence, statistical power is 80%. With these assumptions, if the number of patients who achieve a confirmed response is ≥ 2, then this would allow the rejection of the null hypothesis.

Initially, 15 patients were to be included in the first stage. If at least one confirmed response occurred in the first 15 assessable patients, recruitment would continue up to 25 assessable patients. Two of the first 15 patients had confirmed partial response (PR) to lurbinectedin treatment. Recruitment continued up to 21 patients while evaluating response in the first 15 patients. As the six responses observed in these 21 patients surpassed the threshold of ≥2 confirmed responses established in the statistical hypothesis, no more patients were enrolled.

Descriptive statistics were used. Non-continuous variables are described in frequency tables using counts and percentages. Continuous variables are described by median, minimum and maximum. Binomial exact estimates and 95% confidence intervals (CIs) were calculated for the evaluation of the main endpoint (ORR). The Kaplan–Meier method was used to analyze DoR, PFS and OS. SAS software (SAS Institute, Cary, NC) was used to generate statistical outputs.

## Results

### Patient characteristics

Twenty-one patients were treated with lurbinectedin between 8 January 2016 (first patient enrolled) and the cut-off for the final analysis (16 November 2020). Patients were female (100.0%), white (85.7%), with ECOG PS 0-1 (95.2%) and with a median age of 45 years (range 29-73 years; 9.5% were ≥ 65 years old) (Table 1). Most patients had ductal carcinoma histology (85.7%) and 76.2% had hormone receptor (HR)+ disease. Triple-negative disease was observed in 19.0% of patients and HER2+ in 9.5%. *BRCA1* and *BRCA*2 were reported in 47.6% and 52.4% of patients, respectively. The median number of sites involved at baseline was 2 (range 1-4), with 42.9% of patients having ≥3 disease sites. Bone (*n* = 14; 66.7%), lymph nodes (*n* = 13; 61.9%), liver (*n* = 9; 42.9%) and lung (*n* = 7; 33.3%) were the most common disease sites. Nineteen patients (90.5%) had previously undergone surgery: curative (76.2%) and palliative (33.3%). Prior external radiotherapy had been administered to 18 patients (85.7%). The patients had received a median of two prior lines of chemotherapy for advanced disease (range 0-3 lines). The most common prior agents were paclitaxel (71.4%), cyclophosphamide (61.9%), capecitabine (42.9%) and carboplatin (42.9%). Endocrine therapy had been given to 14 patients (66.7%). Prior PARPi had been administered to 5 patients (23.8%; two of them with time to progression < 6 months), and prior platinum to 10 patients (47.6%; seven of them with platinum-free interval < 6 months). Response rate observed with last prior therapy line was 14.3%.

**Table 1:** Baseline characteristics of the patients (*n* = 21)

	*n*	%
Age: median (range), years	45 (29-73)	
Race		
White	18	85.7
Other^a^	3	14.3
ECOG PS status		
0-1	20	95.2
2	1	4.8
BSA: median (range), m^2^	1.7 (1.5-2.1)	
Albumin: median (range), g/dl	4.1 (3.0-5.0)	
Histological type		
Ductal	18	85.7
Lobular	2	9.5
Unspecified	1	4.8
Hormonal status		
HR+^b^ and HER2+	1	4.8
HR+^b^ and HER2−	15	71.4
HR− and HER2+	1	4.8
Triple negative	4	19.0
*BRCA*		
*BRCA*1	10	47.6
*BRCA*2	11	52.4
No. of sites at baseline: median (range)	2 (1-4)	
≥3 sites	9	42.9
Most common sites of disease at baseline^c^		
Bone	14	66.7
Lymph nodes	13	61.9
Liver	9	42.9
Lung	7	33.3
Bulky disease (one lesion >50 mm)	3	14.3
Prior therapy		
Surgery	19	90.5
Radiotherapy (external)	18	85.7
No. of prior chemotherapy lines for advanced disease: median (range)	2 (0-3)	
Most common prior agents		
Taxanes	20	95.2
Pyrimidine analogues	17	81.0
Anthracyclines	14	66.7
Nitrogen mustard analogues	13	61.9
Platinum compounds	10^d^	47.6
Endocrine therapy	14	66.7
Monoclonal antibodies^e^	6	28.6
PARP inhibitors	5^f^	23.8
Best response to last therapy		
CR	1	4.8
PR	2	9.5
SD	6	28.6
PD	7	33.3
Unknown/not available	5	23.8

Data shown are *n* (%) of patients except for median (range).

BSA, body surface area; CR, complete response; ECOG PS, Eastern Cooperative Oncology Group performance status; HR, hormone receptor; HER2, human epidermal growth factor receptor 2; PARP, poly (ADP-ribose) polymerase; PD, disease progression; PR, partial response; SD, stable disease.

a Patients recruited in France had no race available because of specific ethical requirements in these countries.

b Sixteen patients (76.2%) had overexpressed estrogen or progesterone receptor (HR+) in the tumor.

c Other less common sites included pleura (*n* = 3), breast (*n* = 2) and central nervous system, mediastinum and spinal cord (*n* = 1 each).

d Platinum-free interval (time lapse from last platinum dose until disease progression) was <6 months in 7 of these 10 patients.

e Trastuzumab (*n* = 3), bevacizumab (*n* = 2), pertuzumab (*n* = 2), trastuzumab emtansine (*n* = 1) and monoclonal antibody (not determined).

f Olaparib (*n* = 2), niraparib (*n* = 1), talazoparib (*n* = 1) and veliparib (*n* = 1). Time to progression < 6 months in two of these five patients.

### Lurbinectedin treatment

A total of 188 cycles were administered to the 21 treated patients. The median number of cycles per patient was 6 (range 1-24 cycles), with 61.9% of patients having received ≥5 cycles. The median relative dose intensity was 98.5% (range 62.2%-103.6%).

### Efficacy results

**Figure 1:**
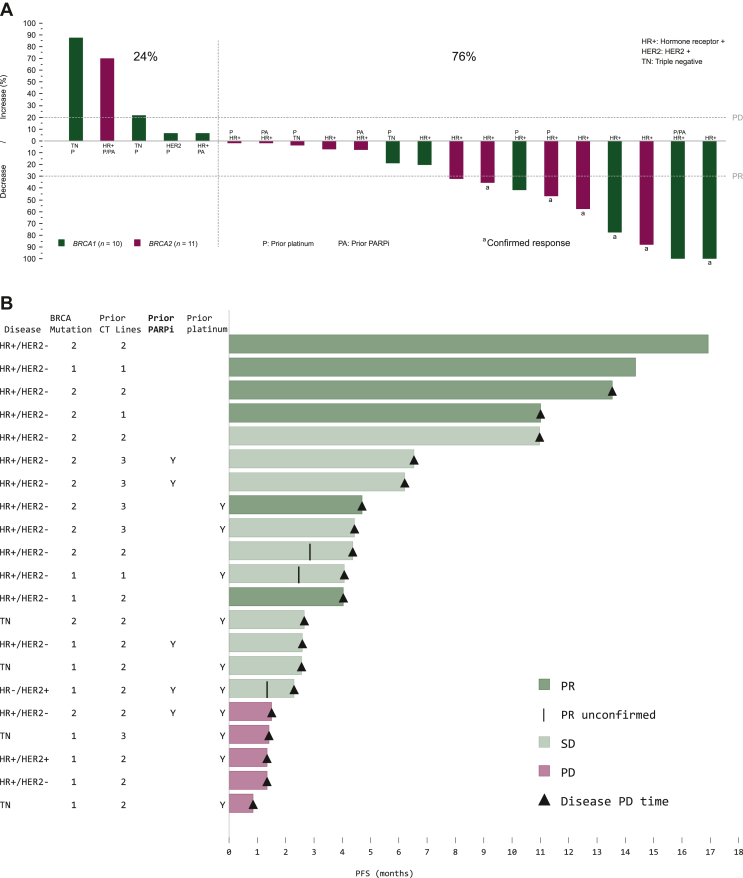
Changes in target lesion size and progression-free survival with lurbinectedin treatment in patients with germline BRCA1/2 metastatic breast cancer. (A) Waterfall plot showing maximum variation of target lesion size with lurbinectedin in patients with germline *BRCA*1/2 metastatic breast cancer. (B) Swimmer plot showing progression-free survival. Each bar represents a patient with germline *BRCA*1/2 metastatic breast cancer treated with lurbinectedin (*n* = 21). CT, chemotherapy; HR, hormone receptor; HER2, human epidermal growth factor receptor 2; PARPi, poly (ADP-ribose) polymerase inhibitor; PD, progressive disease; PFS, progression-free survival; PLAT, platinum; PR, partial response; SD, stable disease; TN, triple negative.

All 21 treated patients were assessable for efficacy. Confirmed PR was observed in six patients; these patients had received a median of two prior advanced chemotherapy lines. Therefore, ORR was 28.6% (95% CI 11.3% to 52.2%). SD was observed in 10 patients (47.6%), including 3 patients with unconfirmed response in a subsequent tumor assessment [ORR unconfirmed = 42.9% (95% CI 21.8% to 66.0%)]. Six patients with SD (28.6%) reached SD ≥ 4 months. (Table 2). Overall, 76.2% of patients had reduction in target lesions during the treatment period (Figure 1A).

**Table 2:** Efficacy results with lurbinectedin treatment in patients with germline *BRCA*1/2 metastatic breast cancer (*n* = 21 assessable patients)

RECIST responses	(*n*, %)
PR	6 (28.6%)
SD^a^	10 (47.6%)
SD ≥ 4 months	6 (28.6%)
PD	5 (36.8%)
ORR, % (95% CI)	28.6% (11.3% to 52.2%)
*BRCA1/BRCA2*	36.4%/20.0%
Clinical benefit rate^b^ (95% CI)	57.1% (34.0% to 78.2%)
Disease control rate^c^ (95% CI)	76.2% (52.8% to 91.8%)
DoR	
Median, months (95% CI)	8.6 (2.9-nr)
DoR at 6 months, % (95% CI)	50.0% (10.0% to 90.0%)
PFS	
Median, months (95% CI)	4.1 (2.3-6.5)
PFS at 6 months, % (95% CI)	33.3% (13.2% to 53.5%)
OS	
Median, months (95% CI)	16.1 (8.7-nr)
OS at 12 months, % (95% CI)	58.1% (35.9% to 80.2%)

CI, confidence interval; DoR, duration of response; OS, overall survival; nr, not reached; PD, disease progression; PFS, progression-free survival; PR, partial response; SD, stable disease.

a Includes three patients with PRs that were not confirmed in a second tumor assessment conducted at least 4 weeks later.

b Clinical benefit rate = PR + SD ≥ 4 months.

c Disease control rate = PR + SD.

Median DoR was 8.6 months (95% CI 2.9 months-upper limit not reached). Clinical benefit rate (PR + SD ≥ 4 months) and disease control rate (PR + SD) were 57.1% (95% CI 34.0% to 78.2%) and 76.2% (95% CI 52.8% to 91.8%), respectively (Table 2). The main characteristics of the patients who achieved clinical benefit (six with PR and six with SD ≥ 4 months) are shown in Table 3. Nine of the 12 patients with clinical benefit had *BRCA*2 mutations (four with PR, five with SD ≥ 4 months); the other three patients had *BRCA*1 mutations (two with PR, one with SD ≥ 4 months). All 12 patients had tumors which were HR+ and HER2−. Regarding prior therapies, two patients had received prior PARPi (both with SD ≥ 4 months) and three patients had been given prior platinum compounds (one PR, two SD ≥ 4 months). No objective response was observed among patients who had received prior PARPi.

**Table 3:** Characteristics of patients with germline *BRCA*1/2 metastatic breast cancer and clinical benefit (CR or PR or SD ≥ 4 Months) with lurbinectedin treatment

Baseline characteristics							Lurbinectedin treatment characteristics						
Age/ECOG PS (years)	Histology type/*BRCA* mutation status	Hormone status	No. of all prior lines/prior chemotherapy lines	Prior PARPi /platinum compounds	Last therapy/best response to last therapy	TTP to last therapy (months)	Cycles received	Location sites	Sum of target lesions at baseline (mm)	% reduction in target lesions	DoR (months)	PFS (months)	OS (months)
Patients with partial response													
51/0	Lobular/*BRCA1*	HR+/HER2−	2/2	No/No	Paclitaxel/SD	6.5	6	Lymph nodes and pleura	54	77.7	2.9	4.0	4.1+
50/2	Ductal/*BRCA2*	HR+ HER2−	3/1	No/No	Fulvestrant and palbociclib/SD	6.5	24	Skin/sub-cutaneous	19	57.8	15.6+	16.9+	17.1+
67/0	Ductal/*BRCA1*	HR+/HER2−	3/2	No/No	Capecitabine/UK	5.0	23	Liver	13	100.0^a^	6.3+	14.4+	16.4+
46/0	Ductal/*BRCA2*	HR+/HER2−	2/2	No/No	Capecitabine/UK	4.1	16	Soft tissue mediastinum	70	35.7	4.8	11.0	26.7
49/0	Ductal/*BRCA2*	HR+/HER2−	4/3	No/Yes	Carboplatin-gemcitabine/UK	6.0	6	Liver	45	75.6	3.5	4.7	11.5
29/0	Ductal/*BRCA2*	HR+/HER2−	2/2	No/No	Capecitabine/CR	17.1	19	Lymph nodes	33	87.8	12.3	13.5	46.9+
Patients with stable disease ≥ 4 months													
45/0	Ductal/*BRCA2*	HR+/HER2−	5/3	Yes/No	Talazoparib/PD	2.1	12	Lymph nodes	52	1.9	NA	6.2+	25.7+
57/0	Ductal/*BRCA2*	HR+/HER2−	3/2	Yes/No	Niraparib/SD	27.6	9	Liver	26	7.6	NA	6.5	20.3+
42/0	Lobular/*BRCA2*	HR+/HER2−	4/3	No/Yes	Carboplatin-trastuzumab-letrozole/PR	11.3	6	Lung/lymph node/skin-subcutaneous	55	1.8	NA	4.4	8.7
43/0	Ductal/*BRCA2*	HR+/HER2−	3/3	No/No	Capecitabine/PR	16.6	8	Liver	53	32.0^b^	NA	4.4	16.1
73/0	Unspecified/*BRCA2*	HR+/HER2−	4/2	No/No	Letrozole/PD	1.7	16	Skin/sub-cutaneous	14	0.0	NA	11.0	20.9+
45/1	Ductal/*BRCA1*	HR+/HER2−	2/1	No/Yes	Gemcitabine-carboplatin/NA	10.4	7	Lymph node/ subpleural	43	41.9^b^	NA	4.1	9.9^b^

CR, complete response; DoR, duration of response; ECOG, Eastern Cooperative Oncology group; HR, hormone receptor; HER2, human epidermal growth factor receptor 2; NA, not available/not applicable; OS, overall survival; PD, disease progression; PFS, progression-free survival; PARPi, poly (ADP-ribose) polymerase inhibitor; PR, partial response; PS, performance status; SD; stable disease; TTP, time to progression; UK, unknown.

a Persistence of non-target lesions.

b The patient had PR that was not confirmed in a subsequent tumor assessment.

Median PFS was 4.1 months (95% CI 2.3-6.5 months). Details per patient are shown in Figure 1B. With a median follow-up of 19.5 months and a censoring rate of 47.6%, median OS was 16.1 months (95% CI 8.7 months-upper level not reached) (Table 2).

Sixteen patients (76.2%) received further antitumor medical therapy (with no objective response), nine patients (42.9%) received further radiotherapy and three patients (14.3%) underwent surgery after lurbinectedin. The most common agents received were eribulin (*n* = 7; 33.3%) and vinorelbine (*n* = 5; 23.8%).

### Safety results

All 21 treated patients were assessable for safety (Table 4). The most common treatment-related AEs were general (mainly fatigue: 38.1% of patients), gastrointestinal (nausea: 61.9%, vomiting: 23.8% and constipation: 19.0%), and metabolism and nutrition disorders (mainly decreased appetite: 14.3%). These AEs were mostly grade 1/2. Treatment-related grade 3/4 AEs and laboratory abnormalities regardless of the relationship were hematological disorders, including leukopenia (28.6%) and neutropenia (42.9%: grade 4, 23.8%: with no cases of febrile neutropenia), fatigue (4.8%), increased weight (4.8%) and increased transaminases (9.5%). Four patients (19.0%) received G-CSFs secondary prophylaxis or therapeutic for neutropenia. No treatment-related serious AEs were reported.

**Table 4:** Most common laboratory abnormalities and treatment-related adverse events (≥10% of patients or grade ≥3) in patients with germline *BRCA*1/2 metastatic breast cancer (*n* = 21)

	NCI-CTCAE grade							
	Grade 1-2		Grade 3		Grade 4		Total	
	*n*	%	*n*	%	*n*	%	*n*	%
Hematological abnormalities (regardless of relationship)								
Anemia	20	95.2	—	—	—	—	20	95.2
Leukopenia	12	57.1	6	28.6	—	—	18	85.7
Neutropenia^a^	7	33.3	4	19.0	5	23.8	16	76.2
Thrombocytopenia	10	47.6	—	—	—	—	10	47.6
Biochemical abnormalities (regardless of relationship)								
Creatinine increased^b^	18	85.7	—	—	—	—	18	85.7
ALT increased	15	71.4	2	9.5	—	—	17	81.0
AST increased	14	66.7	2	9.5	—	—	16	76.2
GGT increased	10	47.6	4	19.0	1	4.8	15	71.4
AP increased	10	47.6	1	4.8	—	—	11	52.4
CPK increased	4	19.0	—	—	—	—	4	19.0
Treatment-related adverse events								
Nausea	13	61.9	—	—	—	—	13	61.9
Fatigue	7	33.3	1	4.8	—	—	8	38.1
Vomiting	5	23.8	—	—	—	—	5	23.8
Constipation	4	19.0	—	—	—	—	4	19.0
Decreased appetite	3	14.3	—	—	—	—	3	14.3
Weight increased	—	—	1	4.8	—	—	1	4.8

AP, alkaline phosphatase; ALT, alanine aminotransferase; AST, aspartate aminotransferase; CPK, creatine phosphokinase; GGT, gamma glutamyltransferase; NCI-CTCAE, National Cancer Institute-Common Terminology Criteria for Adverse Events v.4.

a No cases of febrile neutropenia were observed.

b Version 4.0 of NCI-CTCAE grades creatinine increases from baseline, even if creatinine values remain normal.

Overall, 4.2% of cycles had dose delay due to treatment-related reasons (grade 2/3 neutropenia) in five patients (25.0%). Dose was reduced due to treatment-related reasons in 6.6% of cycles in seven patients (35.0%); the most common cause was neutropenia (grade 2, 3 or 4). Of note, the protocol stated that in case of grade 4 neutropenia, lurbinectedin dose had to be reduced instead of continuing at the same dose with G-CSF prophylaxis.

Most patients (*n* = 19; 90.5%) discontinued treatment due to disease progression. Two of the 21 patients were receiving lurbinectedin treatment at the end of study date and they continued this therapy after the study under compassionate use.

Eleven deaths occurred during the study; all of them were due to progression of the patient’s underlying malignant disease.

## Discussion

This cohort from a phase II exploratory basket study included 21 patients with germline *BRCA*1/2 metastatic breast carcinoma treated with lurbinectedin. The ORR was 28.6% (95% CI 11.3% to 52.2%) in patients who had received a median of two prior chemotherapy lines for advanced disease. These results (six confirmed PRs) were above the threshold of ≥2 confirmed responses established in the statistical hypothesis and the study met its primary endpoint. The results of this trial, together with those of a previous phase II trial,^14^ show that lurbinectedin is active in this population, especially in patients with deleterious *BRCA*2 mutations. The ORR was 36.4% (4/11) in patients with *BRCA*2 mutation and 20.0% (2/10) in patients with *BRCA*1 mutation. Trabectedin, a related compound, also showed higher efficacy in *BRCA2* breast cancer patients versus *BRCA1* (ORR 33% versus 9%).^16^ *BRCA2* prevents the formation of RNA–DNA hybrids (R-loops) that occurs during the elongation step of transcription by RNA polymerase II. One hypothesis to explain the differential activity of trabectedin and lurbinectedin observed in *BRCA2*- compared with *BRCA1*-mutated metastatic breast cancer is the concurrence of deficient DNA repair and the formation of R-loops.^14^

The lower response observed here in comparison with that reported previously (41%; 95% CI 28% to 55%)^14^ could be explained by differences in the number of prior chemotherapy lines for advanced disease (1, range 0-3 versus 2, range 0-3) and prior PARPi (24% versus 17%). Furthermore, all patients in the previous phase II study received doses higher than 3.2 mg/m^2^ (3.5 mg/m^2^ or 7.0 mg flat dose, equivalent to 4.0 mg/m^2^). Nevertheless, the median PFS (4.1 months) was similar to that reported previously (4.6 months)^14^ and the overall rate of unconfirmed response was 42.8%. Some limitations of the current study are the small size of the cohort evaluated, the absence of a central laboratory to confirm *BRCA* status and the lack of sampling during the study to perform pharmacodynamic studies.

Lurbinectedin administered at 3.2 mg/m^2^ as a 1-h i.v. q3wk infusion in patients with pretreated germline BRCA1/2 metastatic breast carcinoma demonstrated a predictable and manageable safety profile, with the main toxicity being reversible myelosuppression, nausea/vomiting and fatigue. Overall, the safety profile reported for lurbinectedin in this cohort of patients is consistent with the results observed previously in patients with breast cancer,^14^ but also in patients with other solid tumors, such as ovarian,^17^^,^^18^ Ewing sarcoma^19^ or SCLC.^12^ The 3.2 mg/m^2^ dose resulted in lower incidences of grade 3/4 AEs compared to the previous phase II trial in which higher doses were used^14^: for instance, grade 3/4 neutropenia 43% versus 67%, febrile neutropenia 0% versus 20%, thrombocytopenia 0% versus 20% or fatigue 5% versus 19%.

In conclusion, the current efficacy results in patients with germline *BRCA*-mutated metastatic breast cancer show lurbinectedin as an active and safe agent in this population, especially in patients with *BRCA*2 mutations. These results are in line with findings in a previous phase II trial. Therefore, development of lurbinectedin in this indication is warranted. Pharmacogenomic and molecular analysis may help to select the patient population that could obtain a higher benefit with lurbinectedin treatment.
